# Supplementary figures and images for: An allelic series at the EDNRB2 locus controls diverse piebalding patterns in the domestic pigeon
Source: PLoS Genet. 2023 Oct 20;19(10):e1010880. doi: 10.1371/journal.pgen.1010880 (PMC10588866; doi:10.1371/journal.pgen.1010880)

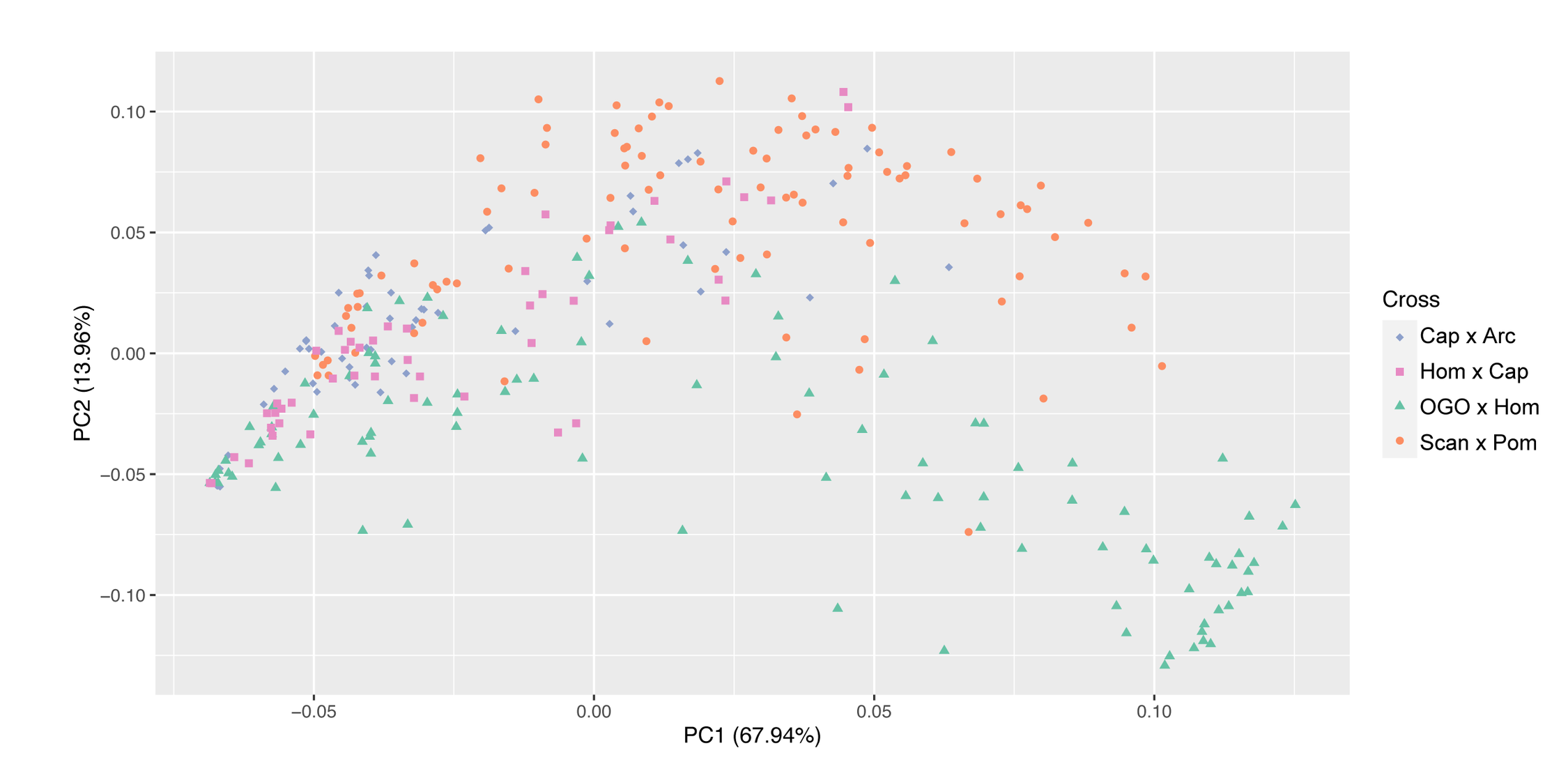

Supplement: S1 Fig — Principal component analysis of plumage phenotyping data for 15 different body regions (shown in Fig 1I) for each cross. Points represent individual F2 birds and are colored by cross. (TIF) [file pgen.1010880.s001.tif]

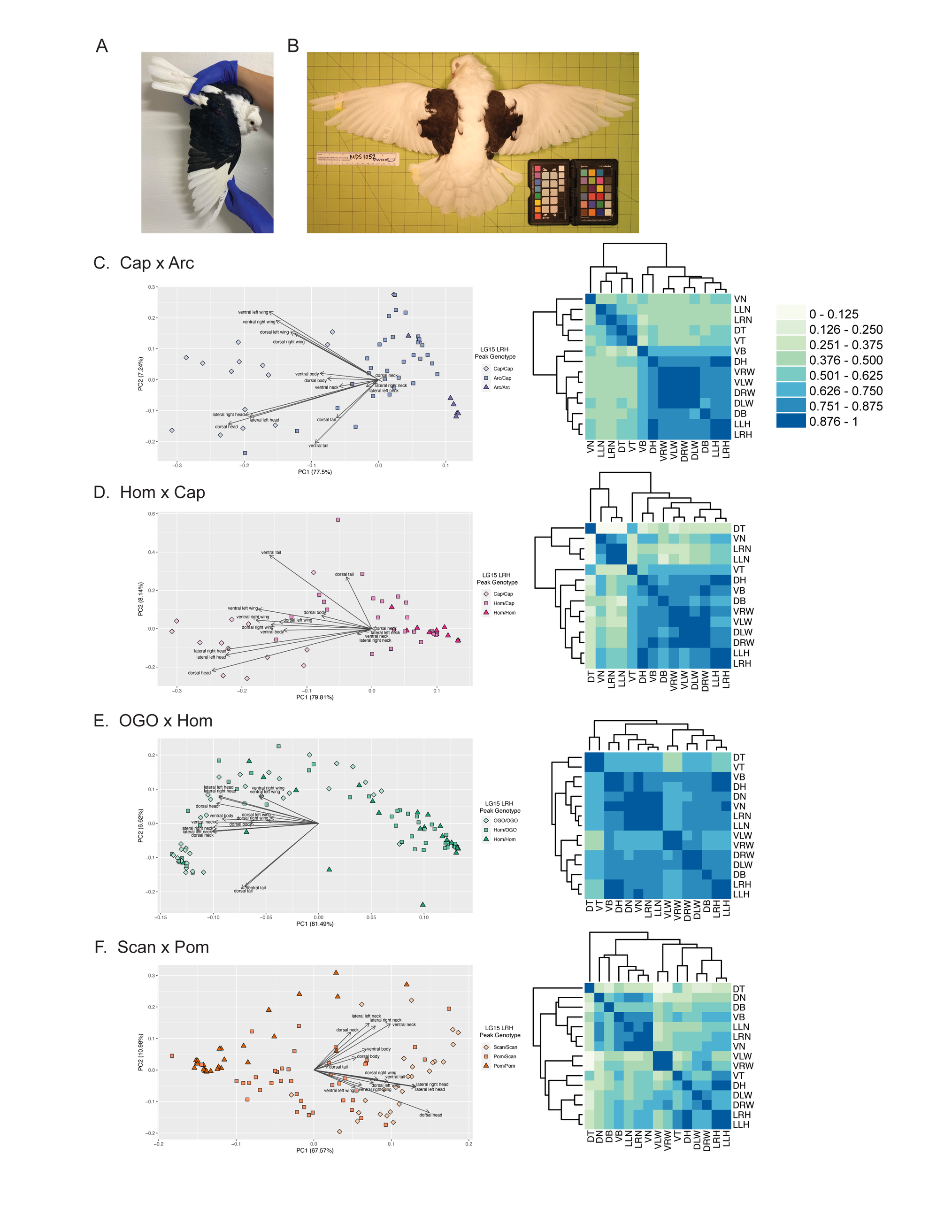

Supplement: S2 Fig — (A) Representative Old German Owl (OGO x Hom founder breed) piebalding phenotype, showing pigmented feathers on the entire wing shield and alula. (B) An example F2 bird from the OGO x Hom cross showing depigmentation on the wing shield that is more extensive than the founder Old German Owl phenotype seen in (A). (C-F) Principal component analysis (left) and correlation diagrams (right) examining relationships between pigmentation across body regions. Arrows show PCA loadings indicating the relative contributions of white plumage in each body region to PC1 and PC2. DT, dorsal tail. DN, dorsal neck. DB, dorsal body. VB, ventral body. LLN, lateral left neck. LRN, lateral right neck. VN, ventral neck. VLW, ventral left wing. VRW, ventral right wing. VT, ventral tail. DH, dorsal head. DLW, dorsal left wing. DRW, dorsal right wing. LRH, lateral right head. LLH, lateral left head. (TIF) [file pgen.1010880.s002.tif]

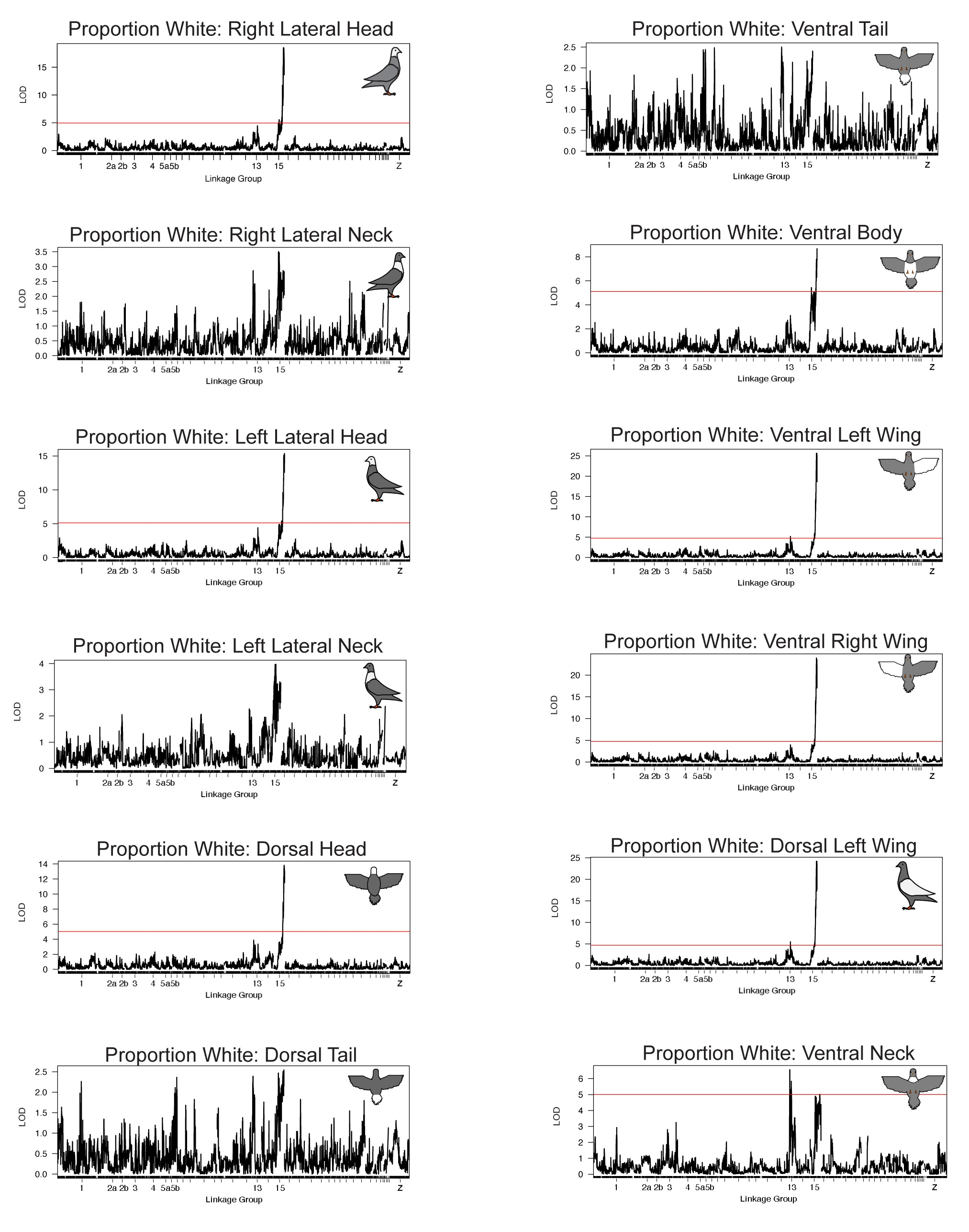

Supplement: S3 Fig — X axis shows linkage groups, Y axis shows LOD score. Red lines indicate the threshold for genome-wide statistical significance. The dorsal neck region was not analyzed in this cross as no F2 birds showed depigmentation in this area. (TIF) [file pgen.1010880.s003.tif]

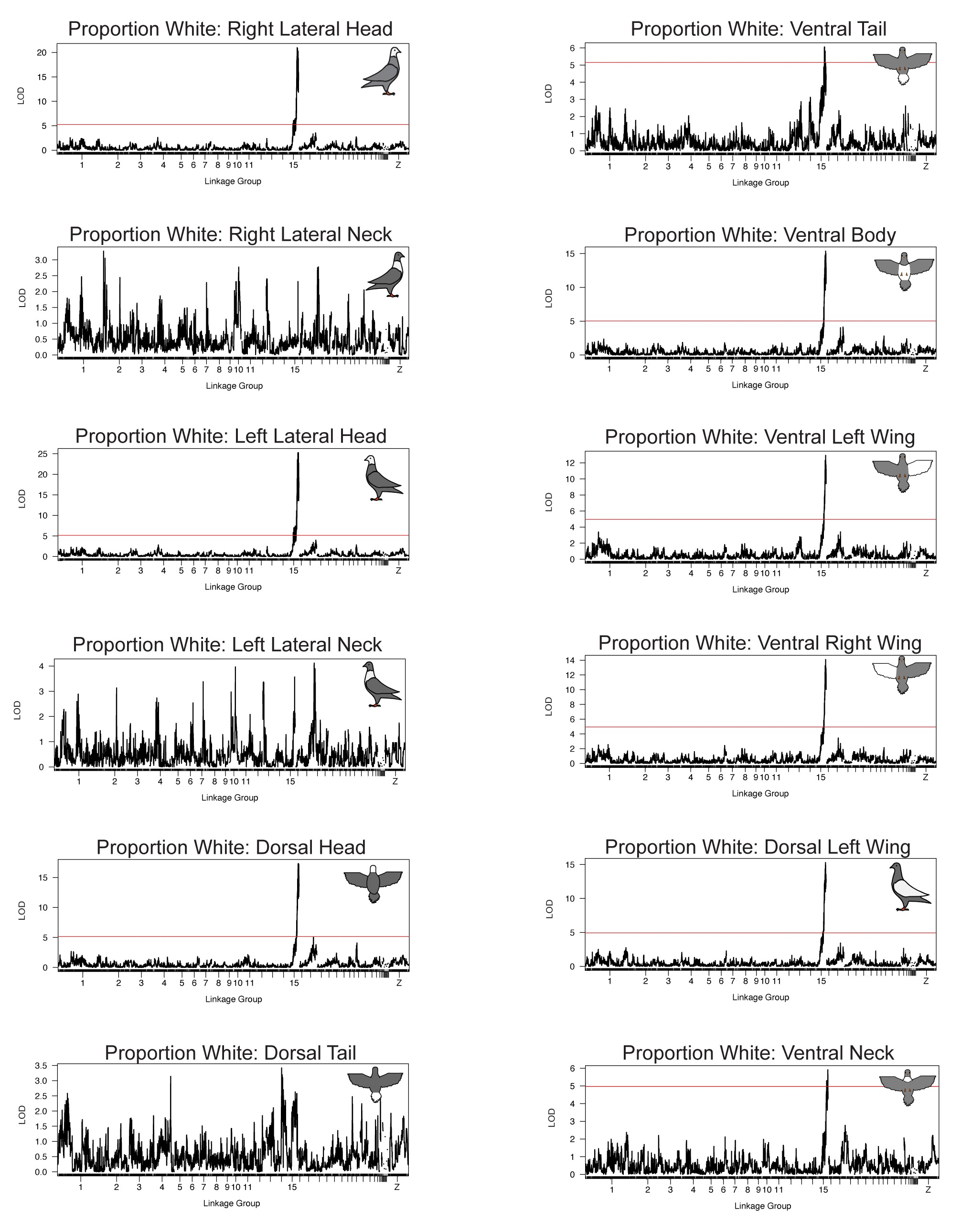

Supplement: S4 Fig — X axis shows linkage groups, Y axis shows LOD score. Red lines indicate the threshold for genome-wide statistical significance. The dorsal neck region was not analyzed in this cross as no F2 birds showed depigmentation in this area. (TIF) [file pgen.1010880.s004.tif]

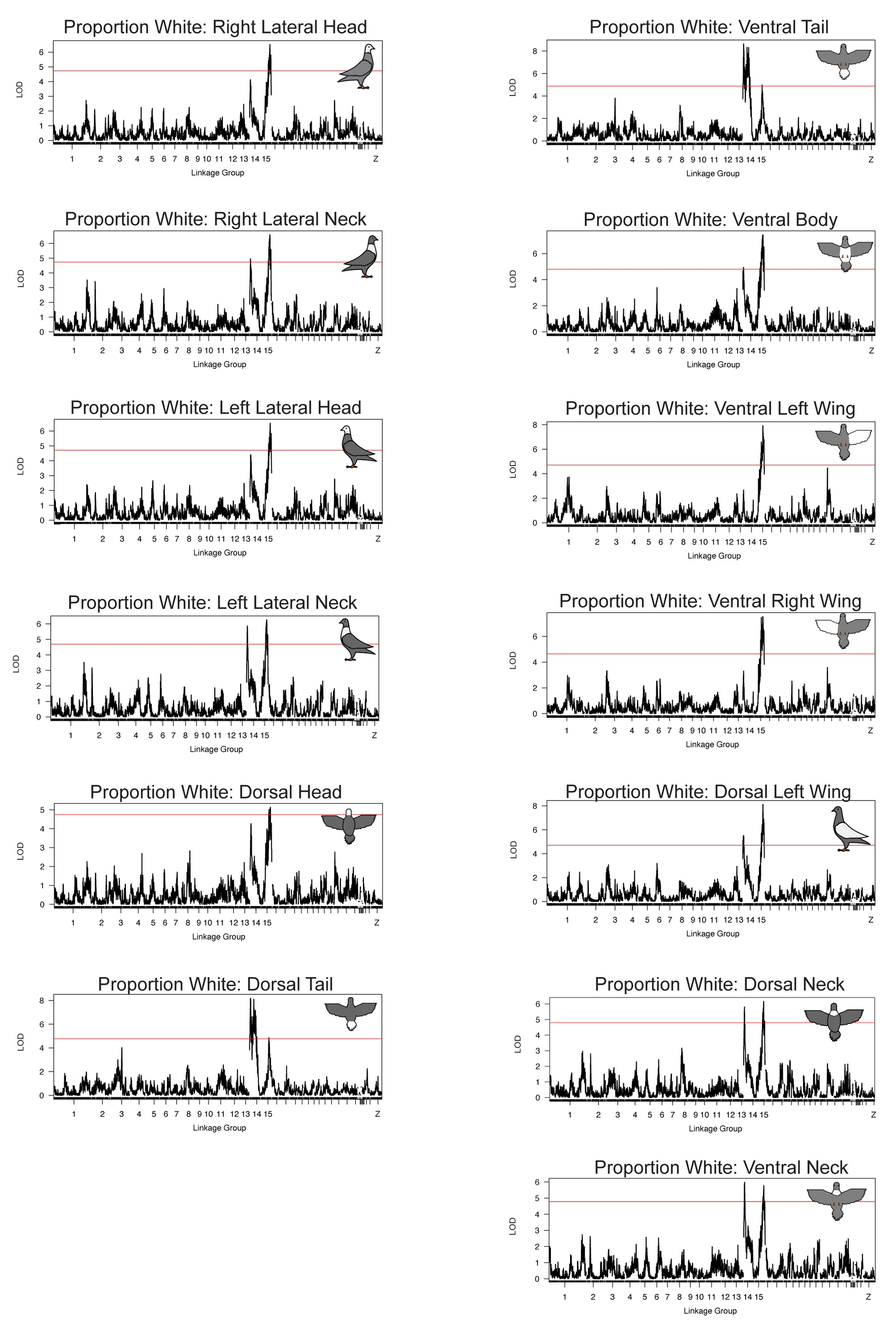

Supplement: S5 Fig — X axis shows linkage groups, Y axis shows LOD score. Red lines indicate the threshold for genome-wide statistical significance. (TIF) [file pgen.1010880.s005.tif]

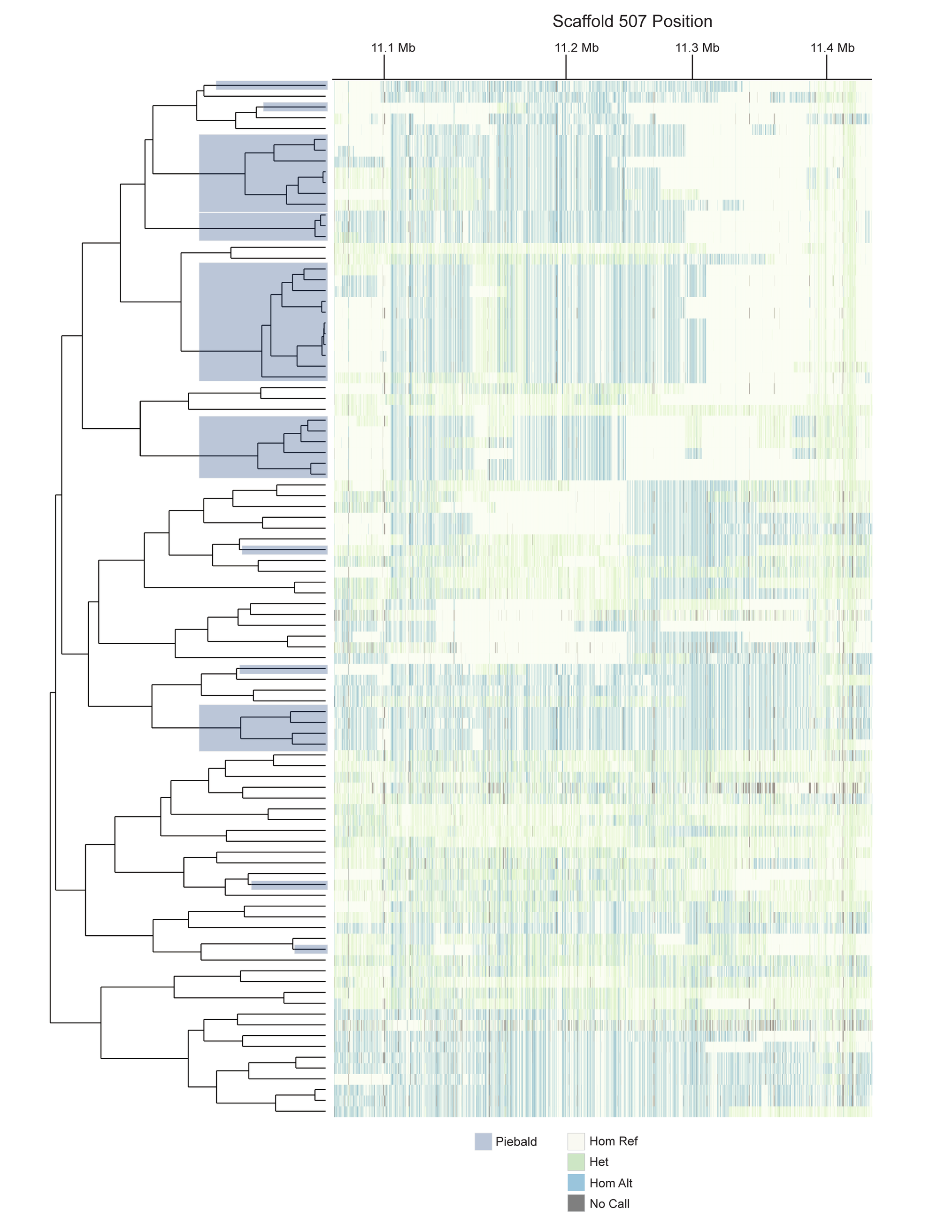

Supplement: S6 Fig — Plot of genotypes within the candidate region identified by pFST. Each row shows an individual bird, and each vertical line a SNP, colored by genotype in relation to the reference genome. Samples are clustered by principal component analysis of genotypes. Samples highlighted in purple are piebald individuals, all others are non-piebald. Hom Ref, homozygous reference allele; Het, heterozygous; Hom Alt, homozygous alternate (non-reference) allele. (TIF) [file pgen.1010880.s006.tif]

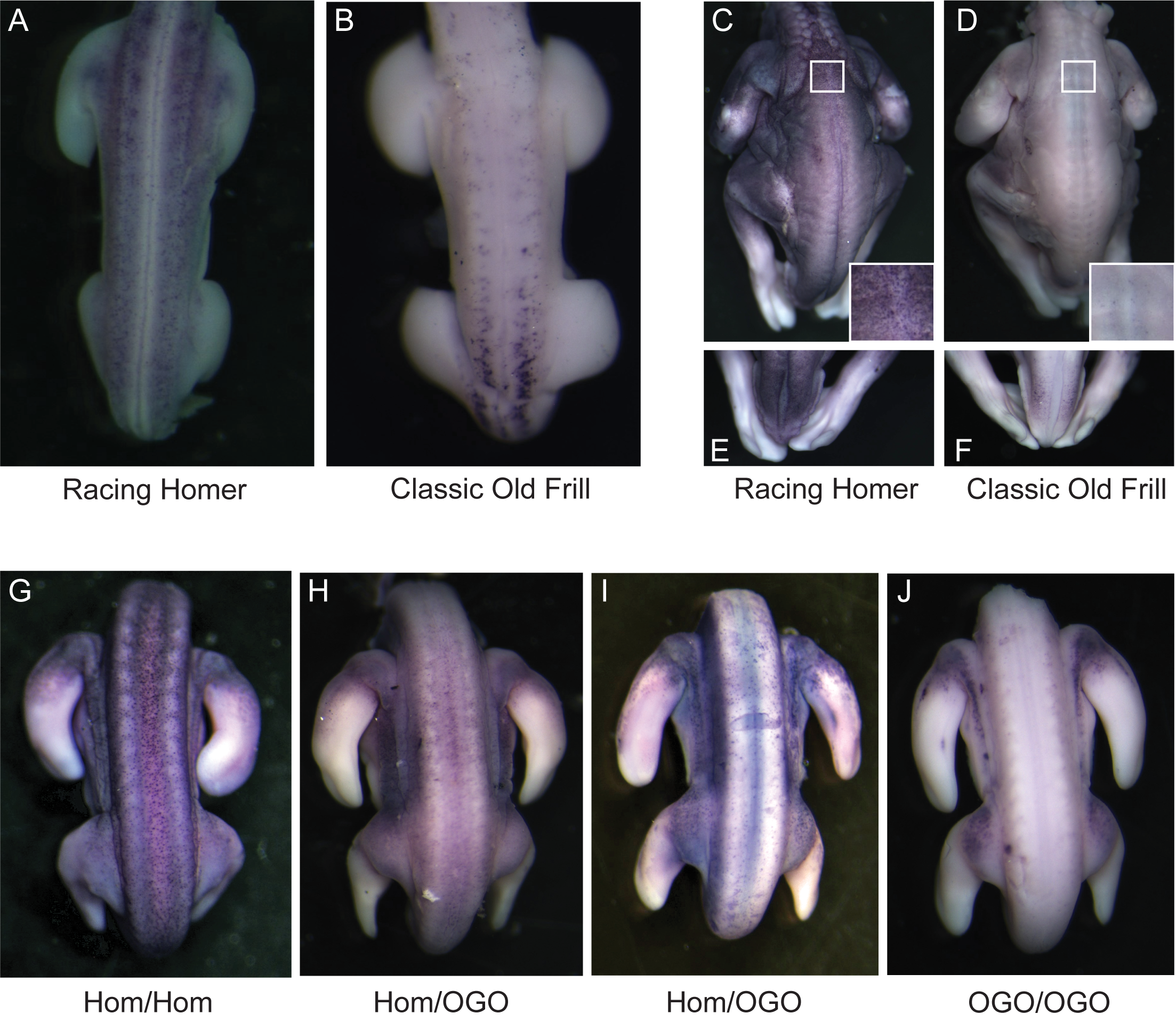

Supplement: S7 Fig — (A,B) Dorsal body of Hamburger-Hamilton stage 24 (HH24) non-piebald Homer (A) and piebald Classic Old Frill (B). (C-H) HH32 non-piebald Homer (C,E) and piebald Classic Old Frill (D,F). C-D, Dorsal body. Insets show the regions indicated by the white squares. E-F, tail bud. (G-J) in situ hybridization for EDNRB2 in representative HH28 Old German Owl x Homer F2 embryos. Genotypes for the peak LG15 QTL marker were determined by Sanger Sequencing and are shown below each embryo. (TIF) [file pgen.1010880.s007.tif]

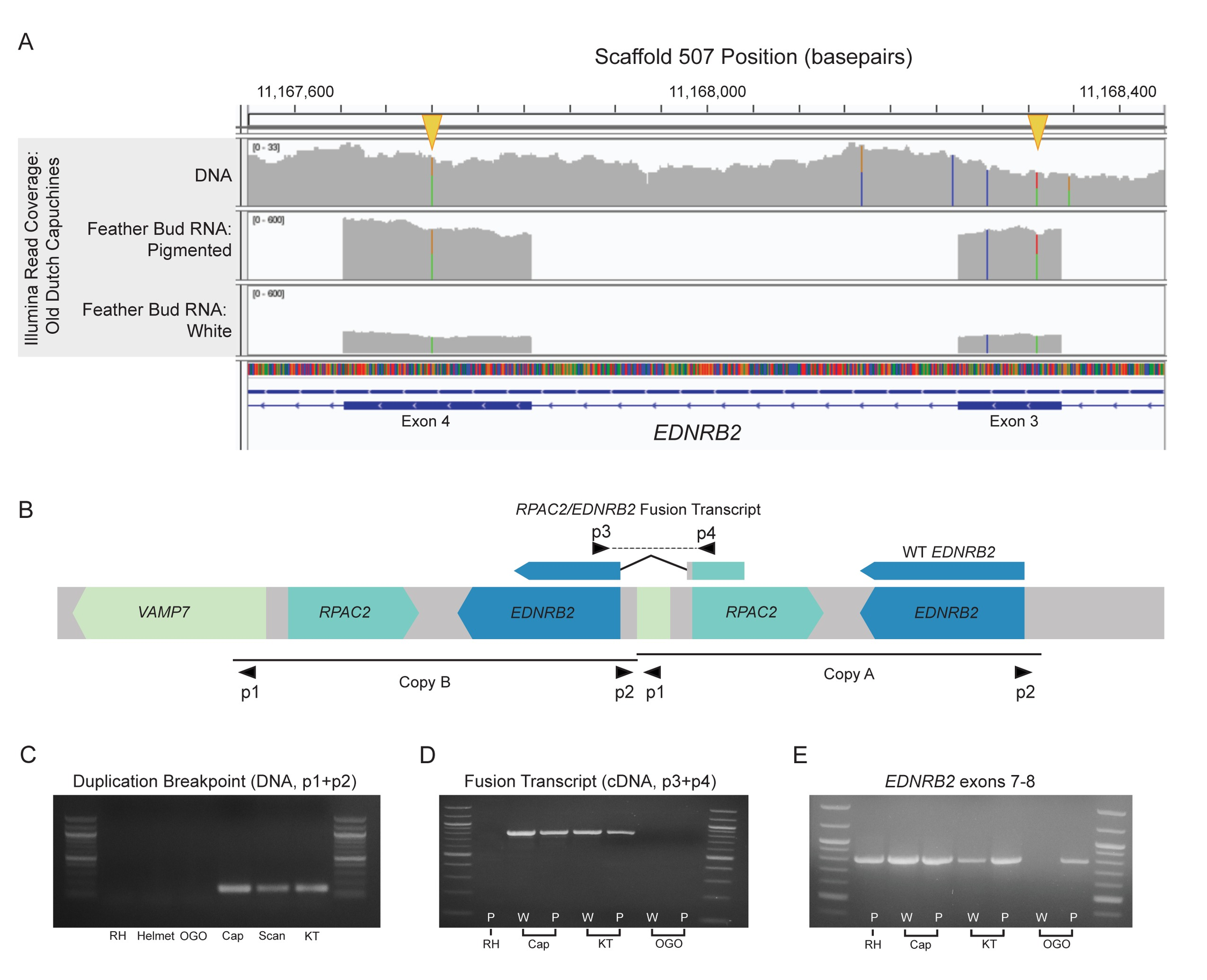

Supplement: S8 Fig — (A) example showing coverage of DNA sequencing (top) and RNA sequencing (middle and bottom) in samples from a representative Old Dutch Capuchine at the EDNRB2 locus. Introns (line) and exons (block) are diagrammed in blue below. Yellow arrows mark two informative SNP sites within EDNRB2 exons. At these sites, colored bars indicate the proportion of reference (orange and red, respectively) or nonreference (green) allele in aligned reads. DNA sequencing indicates that this individual is heterozygous at both sites. RNA-seq from pigmented feathers shows that both alleles are expressed. RNA-seq from white feathers shows expression of only the nonreference (green) allele. Colored bars not marked with arrows are additional SNPs that are not informative for allele-specific expression due to homozygosity or location outside of EDNRB2 exons. (B) Schematic of the predicted structure of the baldhead-associated duplication. The duplicated region includes the 5’ portion of VAMP7 and the entirety of both RPAC2 and EDNRB2. Black triangles below indicate locations of primers. (C) PCR of duplication breakpoint using primers P1 and P2. RH, Racing Homer. OGO, Old German Owl. Cap, Old Dutch Capuchine. Scan, Scandaroon. KT, Komorner Tumbler. (D) RT-PCR using primers P3 and P4 confirms the presence of an RPAC2/ EDNRB2 fusion transcript in white and pigmented feathers from Group 4 Old Dutch Capuchines and Komorner Tumblers, but not other breeds. P, pigmented feather bud sample; W, white feather bud sample. (E) RT-PCR for EDRNB2 confirms the presence of transcripts spliced across exons 7 and 8 in all samples except white feather buds from Old German Owls. (TIF) [file pgen.1010880.s008.tif]

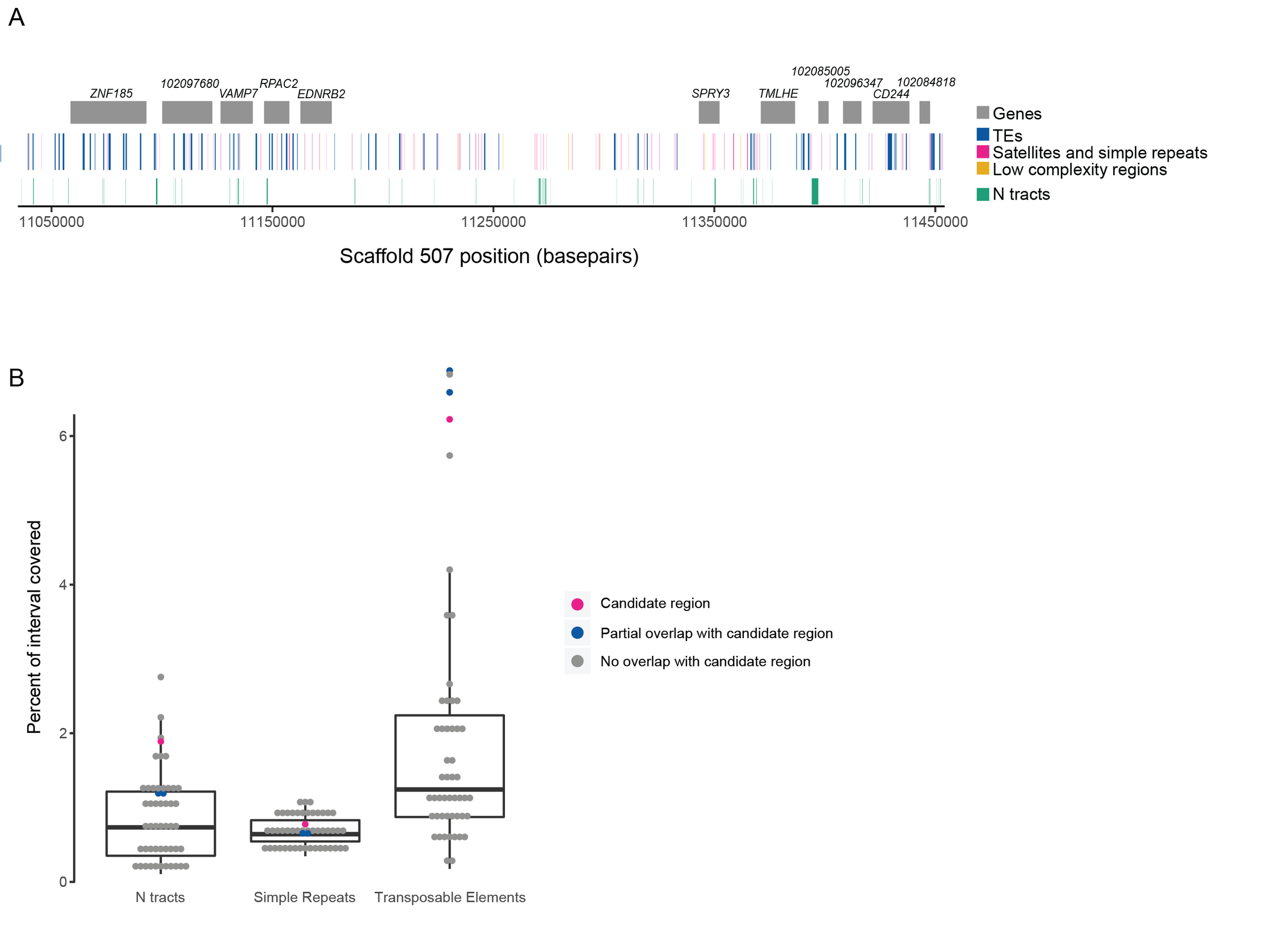

Supplement: S9 Fig — (A) Schematic of the 373 kb piebalding region identified by pFST (See Fig 4). Scaffold position is indicated on the X axis. Gray boxes show approximate locations of genes. The locations of transposable elements (TEs), satellite and simple repeats, low complexity regions, and N tracts are indicated below gene models. (B) Boxplots quantifying coverage by N tracts (left) simple repeats (middle) and transposable elements (right), as annotated by RepeatMasker in the candidate region (pink) and 50 random size-matched regions on scaffold 507 (gray and blue). Blue dots indicate randomly generated regions that partially overlap the candidate region; gray indicates regions with no overlap. Boxes span from the first to third quartile of each data set, with lines indicating the median. Whiskers span up to 1.5x the interquartile range, points beyond whiskers are outliers. (TIF) [file pgen.1010880.s009.tif]
